# Exploring the Association of Hallux Limitus with Baropodometric Gait Pattern Changes

**DOI:** 10.3390/bioengineering12030316

**Published:** 2025-03-19

**Authors:** Natalia Tovaruela-Carrión, Ricardo Becerro-de-Bengoa-Vallejo, Marta Elena Losa-Iglesias, Daniel López-López, Juan Gómez-Salgado, Javier Bayod-López

**Affiliations:** 1 Faculty of Nursing, Physiotherapy and Podiatry, University of Seville, 41009 Seville, Spain; ntovaruela@us.es; 2 Faculty of Nursing, Physiotherapy and Podiatry, Complutense University of Madrid, 28040 Madrid, Spain; ribebeva@ucm.es; 3 Faculty of Health Sciences, Universidad Rey Juan Carlos, 28922 Alcorcón, Spain; marta.losa@urjc.es; 4 Research, Health and Podiatry Group, Department of Health Sciences, Faculty of Nursing and Podiatry, Industrial Campus of Ferrol, Universidade da Coruña, 15403 Ferrol, Spain; 5 Department of Sociology, Social Work and Public Health, Faculty of Labour Sciences, Universidad de Huelva, 21004 Huelva, Spain; salgado@uhu.es; 6 Safety and Health Postgraduate Program, Universidad Espíritu Santo, Guayaquil 092301, Ecuador; 7 Applied Mechanics and Bioengineering Group (AMB), Aragon Institute of Engineering Research (I3A), Centro de Investigación Biomecánica en Red CIBER-BBN, Universidad de Zaragoza, 50009 Zaragoza, Spain; jbayod@unizar.es

**Keywords:** hallux limitus, gait analysis, gait patterns, stance phase gait

## Abstract

**Background:** Hallux limitus (HL) is a condition marked by the restricted dorsiflexion of the first metatarsophalangeal joint, causing pain and functional limitations, especially during the propulsive phase of walking. This restriction affects the gait, particularly in the final phase, and impairs foot stability and support. HL is more common in adults and leads to biomechanical and functional adaptations. The purpose of this study was to investigate the differences in the center of pressure between subjects with hallux limitus and those with healthy feet. **Methods:** A total of 80 participants (40 with bilateral HL and 40 healthy controls) aged 18 to 64 were selected from a biomechanics center at the Universidade da Coruña, Spain. The gait analysis focused on three key phases: initial contact, forefoot contact, and the loading response. Data were collected using a portable baropodometric platform and analyzed using IBM SPSS Statistics 29.0.2.0; statistical significance was set at *p* < 0.05, with a 95% confidence interval. **Results:** The gait analysis indicated that the case group exhibited statistically significant differences, showing lower values in the left foot load response during the foot contact time (77.83 ± 40.17) compared to the control group (100.87 ± 29.27) (*p* = 0. 010) and in the foot contact percentage (*p* = 0. 013) during the stance phase (10.02 ± 5.68) compared to the control group (13.05 ± 3.60). **Conclusions:** Bilateral HL causes subtle gait changes, with individuals showing greater contact time values in the total stance phase versus the control group. Early detection may improve quality of life and prevent complications.

## 1. Introduction

The first metatarsophalangeal joint (IMTFJ) is extremely important in gait patterns—specifically in the terminal stance and pre-swing phases. In these phases, 40% to 60% of the body mass is sustained by the hallux, thus doubling the load that it supports with respect to the lesser toes [1]. The IMTFJ must offer the appropriate dorsiflexion (DF) required to produce the propulsion moment [2,3], and this range of dorsiflexion at the IMTFJ typically averages 58° in normal subjects [2,4]. The concept of hallux limitus (HL) refers to a limitation in the dorsiflexion of the IMTFJ, which usually occurs in 1 out of 40 subjects over the age of 50, making it the main cause of foot arthritis, with an increasing incidence among the elderly population [5]. Senga et al., in their study, determined that the prevalence among people over 50 years of age was 26.7% [6], and, according to Bejarano-Pineda et al., it is as high as 73% in the population with end-stage ankle arthritis [7], with women being twice as likely to suffer from it [8]. The presence of HL is characterized by the appearance of hyperkeratosis in the area of the interphalangeal joint of the hallux, as well as under the head of the second metatarsal [9]. The early stages of HL occur with periods of painful synovitis and slight limitations in IMTFJ dorsiflexion, as osteophytes begin to develop due to arthritic degeneration, resulting in a significant dorsal bony protrusion that may cause friction with footwear and modify the gait [5,10]. When the disease is in a more advanced phase, neuropathic pain, paresthesia, and a positive Tinel sign as a result of the osteophytic compression of the dorsomedial cutaneous nerve are common. The advancement to hallux rigidus is linked to the drastic to total loss of joint functionality, chronic pain, and diminished quality of life [2,11,12]. Although previous studies report that a restriction in the DF movement of the IMTFJ linked to a pronated or highly pronated foot occurs due to biomechanical factors [13,14], there is currently still no agreement on the pathophysiology, cause, or treatment of HL [10,11,15], even though the etiology is considered multifactorial [16,17]

In spite of its clinical importance, the etiology and treatment of HL remain largely unclear [18]. The pathophysiology of HL is not well comprehended, with idiopathy being the most frequently noted etiology [19]. Lengthened metatarsals, hypermobility of the first ray, metatarsus primus elevatus, interphalangeal hallux valgus, being a woman, and having a family history have all been recognized as risk factors [4]. One of the possible etiological factors of HL that has been described is hindfoot eversion, which is associated with a higher degree of DF of the first ray [20].

A decreased range of IMTFJ motion when weight bearing and normal IMTFJ motion when not weight bearing is referred to as functional HL; this results in changes in gait patterns due to the limitation of closed-kinetic-chain joint movement, which restricts function during the terminal phase of gait by the obstruction of the third rocker (propulsive phase) [11,12,21]. The third rocker or forefoot rocker initiates when the heel begins to rise from the surface and ends when the entire foot is lifted from the toe area to begin the swing phase of the foot. At this point, it is important to have good movement in the metatarsophalangeal joints so that no type of support overload occurs in this area [22,23]. The obstruction of the third rocker causes a modification in the sagittal plane of the axis of the first radius; in accordance with the sagittal plane facilitation theory put forward by Dananberg [24], a dynamic limitation in the first MPJ during gait causes a lack of stability, forcing the body to compensate for these biomechanical changes. This leads to the center of mass shifting along the extrinsic and intrinsic structures of the foot to enhance the gait and conclude the propulsion phase [9,22]. Moreover, secondary compensations produced with the aim of improving joint movement to be able to carry out the last phase of walking can cause greater or lesser plantar pressure [12,23,25].

Individuals with HL frequently adopt compensatory gait patterns in order to reduce the discomfort and adjust to restricted mobility [26]. To counteract this limitation, they often shift their weight to the lateral side of the foot, resulting in the overloading of the lateral metatarsal heads, and sometimes push off using the lesser metatarsal heads [27,28], causing increased pressure on the lesser toes and hallux [29], while also showing decreased loading on the IMTFJ [30] and restricted hallux dorsiflexion during the stance and swing phases of gait. Previous pedobarographic studies have shown the increased loading of the lateral plantar areas and lesser metatarsal heads in patients with HL, most likely to avoid pain [31], suggesting compensatory movements in the foot and ankle to facilitate movement and avoid pain during push-off [32]. In addition to its role in locomotion, HL may influence the maintenance of balance. Postural control is a complex mechanism performed by the central nervous system and in which information transmitted by the musculoskeletal system is very important. The feet act as a link between the ground and the body; therefore, any alteration in them can influence the transmission of the forces required to maintain balance [33].

The center of pressure (COP) is described as the point that emerges from the aggregation of the ground reaction forces exerted on the plantar surface of the foot. The COP movement is recognized as an indicator of neuromuscular control while standing and walking and is thus suitable for the assessment of balance control, foot functionality, and the effectiveness of treatment [34]. Through the use of a force platform or pressure platform, this point can be calculated, and the path described by its movement during the test can be followed [31,35]. Despite this, there are aspects that are not clear in people with and without HL, such as the walking pattern and the area of the foot in contact with the surface (percentage), the duration in the foot contact area (milliseconds), and the frames within the foot area (images per second). Consequently, the aim of our research was to investigate the COP variations in subjects with and without HL. We proposed the hypothesis that participants with HL would exhibit an increase in the foot contact duration compared to normal foot contact without HL, as well as a medial shift in the COP during the stance phase.

## 2. Materials and Methods

### 2.1. Design and Sample

This research was carried out as a case–control study following the Strengthening the Reporting of Observational Studies in Epidemiology (STROBE) guidelines [36]. The participants were recruited through a consecutive non-random sample design in a biomechanical center at the Universidade da Coruña (Spain). The study was approved by the Universidade da Coruña Ethics Committee (consent no. 2024-0033), complying with the guidelines of the Declaration of Helsinki and Organic Law 3/2018 on the protection of personal data and guarantee of digital rights [37].

The sample was composed of 80 subjects (11 men and 69 women) aged 21–39 years (mean: 25.22 ± 4.45 SD). The case group was represented by 40 subjects that had bilateral HL and the control group by 40 subjects with healthy common feet.

For the HL group, the following inclusion criteria were taken into account: subjects aged between 18 and 64 years old, without diseases, not having had any surgery or trauma in the lower limbs, presenting bilateral HL, having signed the informed consent form, and participating in the project in all its phases. For the control group, the inclusion criteria were as follows: being between 18 and 64 years old, having no past surgical or traumatic history in the lower limbs, having feet that were bilaterally neutral, having completed the informed consent form, and agreeing to take part in all stages of the project. The exclusion criteria for both groups were as follows: subjects under 18 years of age and over 64 years of age, those with foot discomfort, patients on medication that could affect the final results of the study, pregnancy or breastfeeding, subjects with musculoskeletal disorders or neurological diseases, and individuals who either did not sign the consent form or were unable to comprehend the study guidelines.

### 2.2. Procedure

This research was performed by a skilled podiatrist specializing in biomechanical evaluation. The podiatrist interviewed the participants and provided information about all study details, such as the procedure and duration of the evaluation, as well as informing them that they could withdraw from participation at any time. This detailed explanation contributed to the high acceptance rate. The podiatrist then examined them to ensure that they met the inclusion criteria. The individuals were then weighed and their heights measured to determine their body mass index (BMI). Next, the podiatrist assessed the mobility of the hallux joint [38], and a standard clinical goniometer was employed to confirm the measurements [39]. The evaluation was conducted with the participant in a resting position, where the podiatrist needed to apply a force beneath the I metatarsal head (IMTH).

**Figure 1:**
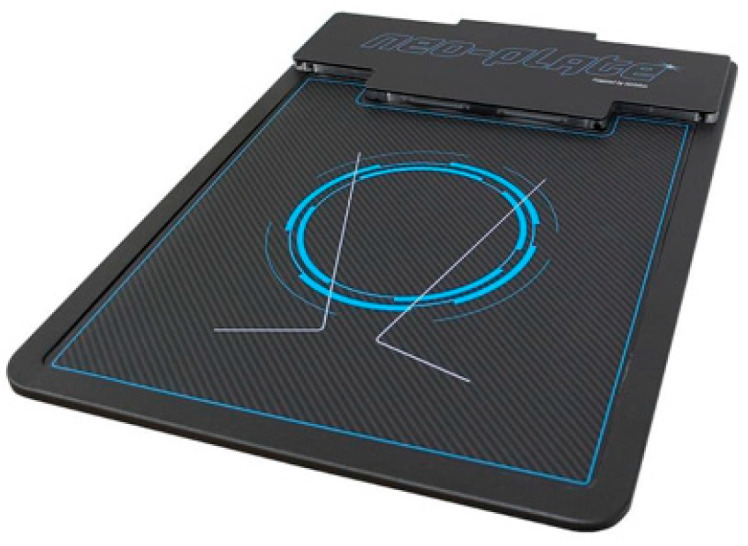
Neo-Plate platform used in this study.

A force similar to the one applied to perform the DF of the hallux was utilized under the IMTH. A negative test (HL-) was deemed to occur if numerous movements were required to attain effective propulsion, and a positive test (HL+) was identified when the force necessary to execute the DF exceeded the one applied under the IMTH. A portable baropodometric platform (Figure 1) (Neo-Plate, Herbitas, Valencia, Spain) [40] was used in this study, according to the established protocol of Becerro de Bengoa Vallejo et al. [41].

All collected data were managed following confidentiality protocols and stored in a secure database. The data were coded to protect the participants’ identities, and only the research team had access to the unidentifiable information.

### 2.3. Dynamic Baropodometric Analysis

A portable pressure platform with resistive sensors was employed with the following characteristics: measurements 40 × 40 cm, thickness of 8 mm, total weight of 4 kg, and 4096 resistive sensors. Measurement was carried out to the nearest 0.01 kPa for each sensor. Autocalibration was conducted before each onset. The Neo-Plate Version 1.12.28.0 for Windows software (Herbitas, Foios, Valencia, Spain) was used to interpret the data, in accordance with the protocol of Becerro de Bengoa Vallejo et al., including a dynamic analysis focusing on the stance pattern of the gait (initial contact phase (ICP); forefoot contact phase (FFCP); flatfoot phase (FFP)), surface contact in foot area (percentage), time in foot contact area (milliseconds), and frames in foot area (images per second) [41]. The dynamics were generated for each foot variable by integrating the surface contact in the foot area (percentage), time in foot contact area (milliseconds), and frames in foot area (images per second).

### 2.4. Statistical Analysis

The G* Power 3.1.9.3 software (Heinrich-Heine-Universität Düsseldorf, Germany) was used to determine the sample size. The input parameters were tail(s) one, an effect size of 0.5, an α error of 0.05, power established by (1-β) of 0.7, and a distribution ratio for two groups established by N2/N = 1.

Statistical analyses were performed with IBM SPSS Statistics 29.0.2.0 for windows (Armonk, NY, USA). The Kolmogorov–Smirnov test was used to check the normality outcomes for all variables in static plantar measurement (*p* < 0.05). Independent t-tests were performed for the variables with a normal distribution. The Mann–Whitney “U” test was used for non-parametric phenomena to contrast groups with and without HL. The independent variables are presented as means, ranges from minimum to maximum, and standard deviation values.

## 3. Results

### 3.1. Sociodemographic Data

The sample consisted of 80 subjects, namely 40 with bilateral HL and 40 healthy controls, of whom 11 were men and 69 were women. The participants ranged in age from 21 to 39 years. A significant proportion of the subjects were overweight, with a BMI of 26.30 ± 5.26 kg/m^2^, showing statistically significant differences (*p* < 0.001). The characteristics of all participants are detailed in Table 1.

### 3.2. Main Outcome Measure Data

As can be seen in the results presented in Table 2, during the gait analysis, the left foot minimum frame FFP was decreased in the subjects with bilateral HL (7.95 ± 4.06) compared to the control group in the left foot (10.30 ± 2.96), with a value of *p* = 0.006. Variations between the groups were noticeable in the time (*p* = 0.010), percentage (*p* = 0.013), and maximum frame in the left foot ICP, with 8.15 ± 4.25 in the case group and 10.50 ± 3.20 in the control group. However, statistically significant differences between the groups were not detected in the gait analysis of the lower left and right limbs.

## 4. Discussion

The main objective of this case–control study was to investigate the COP differences in subjects with and without HL. To the best of our knowledge, this was an innovative study. It is important to emphasize that this was a preliminary exploration aimed at assessing the association between bilateral HL and gait alterations in comparison to healthy individuals. In this study, the protocol used by Becerro de Bengoa in previous research was followed [38,41]. Conducting the discussion was very difficult due to the novelty of this topic, and it was difficult to compare our results with those of other previous studies.

The findings indicated statistically significant variations in the ICP in the left foot in both groups. The results obtained in this research are in agreement with that of Canseco [42], which indicates that the restriction in the range of motion is the most noticeable at pre-swing and toe-off, when the dorsiflexion of the hallux is essential for the propulsion of the limb into the swing phase. These outcomes align with earlier research that suggests that the limited range of motion linked to IMTFJ osteoarthritis disrupts the natural propulsive function of the foot [39,43].

In this study, the group with bilateral HL reduced the contact area in terms of time and percentage. These results can be related to the study by Castro et al., consisting of an analysis of gait parameters in individuals with HL using Optogait [44], which highlighted the existence of alterations in the spatiotemporal aspects of gait compared to the control group. Differences were detected in the step length, the stride length for both the right and left feet, and the loading time. Canseco et al. also found gait alterations in subjects with HL compared to the control group, observing a prolonged support phase and a reduction in walking speed and stride length [42,45,46]. Therefore, according to these results, the presence of HL alters the dynamics of walking and affects its normal development.

In our study, significant differences were observed in the minimum frame in the forefoot contact phase for the left foot. Research has shown that the presence of HL causes greater plantar pressure to be generated under the hallux and therefore an increase in pressure on the rest of the forefoot [12,29,31]. Zammit et al., in their study, indicated that people with IMTFJ osteoarthritis displayed significantly greater maximum forces and peak pressures under the hallux and lesser toes [30].

Morasiewicz et al. conducted a study similar to ours but in subjects with a degenerative ankle injury (osteoarthritis), where they evaluated the change in balance and load distribution in the lower limbs in patients before and after ankle arthrodesis. They observed that the deformity, pain, and reduced range of motion affected the distribution of loads in the lower limbs [47]. In their study, the results showed that, after treatment with arthrodesis, they achieved almost a symmetrical distribution of loads, but with worse results than in the healthy group, and they concluded that this may have been due to the fact that the treatment only acted on the ankle, while, in these subjects, the movement of the toes, knee, and hip is restricted, altering the biomechanics of the neighboring joints. Therefore, long-term alterations of the foot and ankle, such as HL in this case, can lead to the central nervous system adapting to this condition, triggering compensatory mechanisms that can even affect other joints, altering the entire biomechanics of the lower limb and increasing the risk of secondary musculoskeletal problems due to changes in load distribution and joint function. This can limit the conditions for successful treatment if the pathology is detected at an advanced stage, preventing the musculoskeletal system from normalizing both statically and dynamically, thus highlighting the importance of our study and of detecting HL in time in order to prevent future complications.

A portable baropodometric platform was used in this research. Wearable technology, which helps to detect alterations in gait balance that may be related to foot pathologies, has been used in various studies on gait analysis with the aim of assessing body movement in individuals with and without biomechanical alterations. Other authors [44,46], using other different technological systems, have also found similar results, demonstrating that subjects with HL present gait disturbances. Therefore, the findings of these studies corroborate our outcomes, i.e., HL modifies the dynamic characteristics of walking.

Our study has some limitations; for example, the baropodometric platform could only record and identify vertical forces at a frequency of 60 Hz, and it would be interesting to use other frequencies and different forces in the capture and recording of force movement on the foot alone. In our study, only some parameters showed statistically significant differences between the groups, and most of them did not differ between the groups, so the biomechanical alteration observed was minor. Therefore, for future studies, it would also be interesting to assess other joints that may be altered as a consequence of HL. Biomechanical musculoskeletal lower-limb gait model data are of significant interest, since gait disturbances and loss of balance can cause falls, which are important to avoid. Therefore, for future studies, it would be interesting to evaluate other aspects with the aim of improving the gait in these subjects, through the premature compensation of the foot as a preventive measure. This is especially important in elderly subjects in order to avoid the risk of falls and improve the gait, thus enhancing their autonomy and quality of life. In our study, statistical power of 0.70 was used due to the limitations inherent in the available sample size and the characteristics of the studied population. Therefore, we will consider increasing the sample size in future studies to improve this aspect.

However, this study supplies information on a foot disorder for podiatrists and researchers regarding stance phase gait parameters in subjects with HL deformities. Deeper insight into the relationship between HL and modified gait biomechanics could provide the clinician with a more thorough evaluation of the condition as it pertains not only to hallux movement but also to its contribution to lower-extremity functionality. Continued research on this subject is important to facilitate early diagnosis and effective treatment for patients in order to improve their foot health and their general quality of life.

## 5. Conclusions

The existence of bilateral HL causes slight changes in gait analysis when compared to healthy individuals. Participants with bilateral HL, in contrast to the control group, demonstrated a reduced minimum frame FFP on the left foot. Differences in the time, percentage, and maximum frame in the left foot ICP were noted between the two groups, which may be associated with foot deformity. We believe that the early identification of these changes could enhance patients’ quality of life and avert future complications. Therefore, it is necessary to continue with this line of research by analyzing new variables that may be related to the presence of HL.

## Figures and Tables

**Table 1:** Main characteristics of total sample with and without bilateral hallux limitus.

Characteristics	Total Sample (n = 80) Mean ± SD (Range)	Case Group (n = 40) Mean ± SD (Range)	Control Group (n = 40) Mean ± SD (Range)	*p*-Value
Age (years)	25.22 ± 4.45 (21–39)	25.45 ± 4.34 (21–36)	25.00 ± 4.60 (21–39)	0.214 †
Weight (kg)	71.58 ± 13.69 (53–98)	76.75 ± 14.80 (56–98)	66.40 ± 10.30 (53–89)	**<0.002 ** **†**
Height (cm)	165.15 ± 0.08 (150–185)	163.15 ± 0.08 (152–185)	166.15 ± 0.08 (150–185)	0.231 †
BMI (kg/m^2^)	26.30 ± 5.26 (19.00–39.26)	28.58 ± 6.00 (21.08–39.26)	24.02 ± 3.06 (19.00–32.44)	**<0.001 ** **†**
Sex, male/female (%)	11/69 (13.8/86.3)	7/33 (17.5/82.5)	4/36 (10/90)	0.518 ‡
Foot size	39.00 ± 2.26 (36–46)	38.83 ± 1.98 (36–44)	39.18 ± 2.53 (36–46)	0.600 †

Abbreviations: kg, kilogram; cm, centimeter; %, percentage; SD, standard deviation; N, number. † Mann–Whitney U test was used. ‡ Fisher exact test was used. In all analyses, *p* < 0.05 (with a 95% confidence interval) was considered statistically significant (bold).

**Table 2:** Main outcome measurements of gait analysis of total sample with and without bilateral hallux limitus.

Characteristic	Total Sample (n = 80) Mean ± SD (Range)	Case Group (n = 40) Mean ± SD (Range)	Control Group (n = 40) Mean ± SD (Range)	*p*-Value
Left foot FFCP (ms)	241.23 ± 57.22 (148–414)	243.78 ± 45.28 (178–335)	238.67 ± 67.59 (148–414)	0.349 †
Left foot FFCP (%)	33.21 ± 7.34 (20–54)	33.65 ± 6.77 (21–54)	32.78 ± 7.39 (20–48)	0.415 †
Left foot min frame FFCP	51.78 ± 8.71 (32–73)	51.95 ± 9.33 (32–73)	51.60 ± 8.15 (34–70)	0.555 †
Left foot max frame FFCP	76.24 ± 7.46 (58–91)	76.65 ± 7.38 (68–91)	75.82 ± 7.61 (58–89)	0.958 †
Left foot FFP (ms)	419.66 ± 90.39 (275–632)	433.03 ± 104.03 (275–632)	406.30 ± 73.23 (275–593)	0.196 †
Left foot FFP (%)	55.25 ± 8.75 (38–70)	56.33 ± 9.36 (41–70)	54.17 ± 8.06 (38–69)	0.359 †
Left foot min frame FFP	9.13 ± 3.72 (1–17)	7.95 ± 4.06 (1–13)	10.30 ± 2.96 (3–17)	**0.006 ** **†**
Left foot max frame FFP	51.75 ± 8.72 (32–73)	51.90 ± 9.36 (32–73)	51.60 ± 8.15 (34–70)	0.514 †
Left foot ICP (ms)	89.35 ± 36.80 (9–167)	77.83 ± 40.17 (9–128)	100.87 ± 29.27 (29–167)	**0.010 ** **†**
Left foot ICP (%)	11.54 ± 4.96 (1–21)	10.02 ± 5.68 (1–18)	13.05 ± 3.60 (4–21)	**0.013 ** **†**
Left foot min frame ICP	0 ± 0.00 (0–0)	0 ± 0.00 (0–0)	0 ± 0.00 (0–0)	1.000 †
Left foot max frame ICP	9.33 ± 3.92 (1–17)	8.15 ± 4.25 (1–16)	10.50 ± 3.20 (3–17)	**0.009 ** **†**
Right foot FFCP (ms)	261.50 ± 139.24 (148–748)	278.65 ± 173.85 (148–748)	244.35 ± 91.91 (158–455)	0.900 †
Right foot FFCP (%)	35.81 ± 17.56 (20–99)	37.63 ± 22.32 (21–99)	34.00 ± 10.93 (20–58)	0.896 †
Right foot min frame FFCP	49.76 ± 14.96 (1–71)	48.95 ± 18.40 (1–71)	50.58 ± 10.66 (30–68)	0.664 †
Right foot max frame FFCP	76.31 ± 8.41 (54–91)	77.23 ± 7.30 (68–89)	75.40 ± 9.40 (54–91)	0.527 †
Right foot FFP (ms)	404.57 ± 129.36 (0–602)	391.38 ± 157.12 (0–602)	417.77 ± 94.09 (254–561)	0.881 †
Right foot FFP (%)	53.40 ± 15.50 (0–70)	50.88 ± 19.01 (0–68)	55.93 ± 10.57 (34–70)	0.434 †
Right foot min frame FFP	8.63 ± 3.48 (1–18)	9.15 ± 3.32 (1–18)	8.10 ± 3.61 (1–14)	0.090 †
Right foot max frame FFP	49.76 ± 14.96 (1–71)	48.95 ± 18.40 (1–71)	50.58 ± 10.66 (30–68)	0.664 †
Right foot ICP (ms)	84.41 ± 34.42 (9.00–176)	89.58 ± 32.76 (9.00–176)	79.25 ± 35.67 (9.00–138)	0.078 †
Right foot ICP (%)	10.79 ± 4.59 (1–22)	11.50 ± 4.57 (1–22)	10.08 ± 4.54 (1–17)	0.282 †
Right foot min frame ICP	0 ± 0.00 (0–0)	0 ± 0.00 (0–0)	0 ± 0.00 (0–0)	1.000 †
Right foot max frame ICP	8.63 ± 3.48 (1–18)	9.15 ± 3.32 (1–18)	8.10 ± 3.61 (1–14)	0.090 †

Abbreviations: ICP, initial contact phase; FFCP, forefoot contact phase; FFP, flatfoot phase. ms, meters per second; kpa, kilopascal; %, percentage; SD, standard deviation; N, number. † Mann–Whitney U test was used. In all analyses, *p* < 0.05 (with a 95% confidence interval) was considered statistically significant (bold).

## Data Availability

The dataset supporting the conclusions of this article is available upon request to daniellopez@udc.es in the Research, Health and Podiatry Group, Department of Health Sciences, Faculty of Nursing and Podiatry, Industrial Campus of Ferrol, Universidade da Coruña, 15403, Ferrol, Spain.
